# The Dipeptidyl Peptidase-4 Inhibitor Linagliptin Preserves Endothelial Function in Mesenteric Arteries from Type 1 Diabetic Rats without Decreasing Plasma Glucose

**DOI:** 10.1371/journal.pone.0143941

**Published:** 2015-11-30

**Authors:** Salheen M. Salheen, Usha Panchapakesan, Carol A. Pollock, Owen L. Woodman

**Affiliations:** 1 School of Medical Sciences, RMIT University, Bundoora, Victoria, Australia; 2 Kolling Institute of Medical Research, Royal North Shore Hospital, University of Sydney, St Leonards, New South Wales, Australia; University of Missouri, UNITED STATES

## Abstract

The aim of the study was to investigate the effect of the DPP-4 inhibitor linagliptin on the mechanism(s) of endothelium-dependent relaxation in mesenteric arteries from STZ-induced diabetic rats. Both normal and diabetic animals received linagliptin (2 mg/kg) daily by oral gavage for a period of 4 weeks. To measure superoxide generation in mesenteric arteries, lucigenin-enhanced chemiluminescence was used. ACh-induced relaxation of mesenteric arteries was assessed using organ bath techniques and Western blotting was used to investigate protein expression. Pharmacological tools (1μM TRAM-34, 1μM apamin, 100 nM Ibtx, 100 μM L-NNA, 10 μM ODQ) were used to distinguish between NO and EDH-mediated relaxation. Linagliptin did not affect plasma glucose, but did decrease vascular superoxide levels. Diabetes reduced responses to ACh but did not affect endothelium-independent responses to SNP. Linagliptin improved endothelial function indicated by a significant increase in responses to ACh. Diabetes impaired the contribution of both nitric oxide (NO) and endothelium-dependent hyperpolarization (EDH) to endothelium-dependent relaxation and linagliptin treatment significantly enhanced the contribution of both relaxing factors. Western blotting demonstrated that diabetes also increased expression of Nox2 and decreased expression and dimerization of endothelial NO synthase, effects that were reversed by linagliptin. These findings demonstrate treatment of type 1 diabetic rats with linagliptin significantly reduced vascular superoxide levels and preserved both NO and EDH-mediated relaxation indicating that linagliptin can improve endothelial function in diabetes independently of any glucose lowering activity.

## Introduction

Endothelial dysfunction is considered a critical factor in the initiation and development of vascular complications induced by diabetes [1, 2]. Macro- and microvascular complications are presently the main causes of morbidity and mortality amongst diabetic patients in both type 1 and type 2 diabetes mellitus [3]. Endothelial cells play an active role to regulate the basal vascular tone and reactivity of blood vessels in both physiological and pathological conditions, by releasing contracting and relaxing factors in response to stimulating factors such as mechanical forces and neurohumoral mediators [4, 5]. The most important endothelium-derived relaxing factors (EDRFs) are nitric oxide (NO), prostacyclin and endothelium-dependent hyperpolarization (EDH) [6] and we have previously reported that high glucose and diabetes impair the contribution of both NO and EDH to endothelium-dependent relaxation [7, 8].

Dipeptidyl peptidase-4 (DPP-4) is a glycoprotein peptidase broadly expressed in various cell types which displays complex biological actions and has multiple functions [9–12]. DPP-4 inhibitors comprise a new class of blood glucose-lowering drugs for the treatment of type 2 diabetes with advantages of their neutral effect on body weight and low risk of the occurrence of hypoglycemia [13]. DPP-4 inhibitors prolong the half-life of incretins, such as glucagon-like-peptide-1 (GLP-1) and glucagon-induced peptide (GIP), and thus lower blood glucose via improved insulin secretion [9]. Interestingly, previous studies have demonstrated additional beneficial effects of GLP-1 in situations such as in the regulation of endothelial function and cardiac remodeling [14–17] and the DPP-4 inhibitors have been reported to reduce the impairment of cardiac diastolic function in insulin resistant male Zucker obese rats [18], to improve the obesity-related glomerulopathy in Zucker obese rat [19], to ameliorate dysfunction in rat aortic artery in experimental sepsis [20] and to reduce oxidative stress in vascular endothelial cells [21]. We have recently found that acute treatment with linagliptin ameliorates vascular dysfunction in mesenteric arteries exposed to high concentration of glucose (40 mM) demonstrating a beneficial action independently of any glucose lowering effect [8].

We have previously demonstrated that in small arteries diabetes-induced endothelial dysfunction results from the impairment of both NO-mediated and EDH-mediated relaxation, associated with eNOS uncoupling and an increase in Nox2-derived superoxide generation [7]. We have also shown that treatment with 3’,4’-dihydroxyflavonol reduces oxidative stress and improves endothelium-dependent relaxation in type 1 diabetic rats [7]. Therefore, it is of particular interest to examine whether linagliptin, a DPP-4 inhibitor which we have recently demonstrated is able to also act as an antioxidant [8], can alleviate endothelial dysfunction in diabetic vasculature independently of its glucose lowering properties. Importantly this antioxidant activity of linagliptin was not shared by 2 other DPP-4 inhibitors sitagliptin and vildagliptin. Whilst this in vitro finding is of interest the major question remains as to whether linagliptin treatment in vivo can improve endothelial function after several weeks of hyperglycaemia. Thus, the aim of the present study was to examine whether chronic *in vivo* treatment with the DPP-4 inhibitor linagliptin, preserves endothelial function in small mesenteric arteries from type 1 STZ-induce diabetic rats and whether there was an associated reduction in the generation of vascular ROS. Importantly in this study we used a model of type 1 diabetes where any beneficial action of linagliptin on endothelial function was not secondary to a reduction in hyperglycaemia.

## Methods

### Ethics statement

This study and all procedures employed in it were approved by the Animal Experimentation Ethics committee of RMIT University (AEC approval number 1309) and complied with the Australian National Health and Medical Research Council code of practice for the care and use of animals for scientific purposes.

### Induction of type 1 diabetes

Male Wistar rats (~8-weeks old and weighing approximately 200g) were randomly divided into two groups, normal and diabetic. A single injection of streptozotocin (STZ, 50 mg/kg IV) after the rats were fasted overnight was used to induce type 1 diabetes, whereas, rats in the control group received vehicle of a comparable volume (0.1 mol/l citrate buffer, pH 4.5). Blood glucose was monitored and when greater than 25 mmol/l, rats were treated with insulin (3–4 IU s.c., 3 injections/week, Protaphane, Novo Nordisk, NSW, Australia). Blood glucose concentration was measured by a glucometer (Roche, Sydney, NSW, Australia) and glycated haemoglobin (HbA_1c_) was measured using a Micromat HbA_1c_ analyser (Biorad, Sydney, NSW, Australia). Blood samples were collected from the left ventricle at the end of the experimental period.

#### Linagliptin treatment

Six weeks after induction of diabetes, the two groups of rats were further divided into 4 subgroups (normal, normal+linagliptin, diabetic, diabetic+linagliptin) administering either vehicle (1% carboxy methylcellulose) or linagliptin (2 mg/kg) daily by oral gavage for a period of 4 weeks. The rats were administered the last dose of linagliptin at least 24 hours prior to the collection of tissue for experimentation.

### Isolation of mesenteric arteries

Ten weeks after STZ treatment, the rats were killed by asphyxiation with CO_2_ followed by exsanguination. The mesenteric arcade was isolated and collected and immediately placed in ice-cold Krebs bicarbonate solution (4.7 mmol/l KCl, 118 mmol/l NaCl, 1.18 mmol/l MgSO_4,_ 25 mmol/l NaHCO_3,_ 11.1 mmol/l D-glucose and 1.6 mmol/l CaCl_2_) containing the non-selective cyclooxygenase (COX) inhibitor indomethacin (10 μmol/l), to inhibit the production of prostanoids. Small mesenteric arteries (third order branch of the superior mesenteric artery, internal diameter ~300 μm) were isolated, cleared of fat and connective tissue and dissected into rings of about 2 mm in length and then mounted on a Mulvany-Halpern myograph (model 610M, Danish Myo Technology, Aarhus, Denmark). After the rings were mounted, they were allowed to stabilize at zero tension for 15 min before normalization. The blood vessels were stretched to attain an internal pressure comparable to 90% of that of the blood vessel under a transmural pressure of 100 mmHg [22, 23] to determine the passive tension-internal circumference. All experiments were conducted at 37°C in the Krebs solution bubbled with carbogen (95% O_2_ and 5% CO_2_).

#### Investigation of vascular function and reactivity

After equilibration for 30 minutes, blood vessels were subjected to maximum contraction by the addition of an isotonic physiological saline solution containing a high concentration of K^+^ (123 mmol/l, KPSS). In order to investigate the integrity of the endothelium, the vessels were precontracted with phenylephrine (0.1–3 μmol/l) to ~50% of the KPSS response and relaxed with a high single dose of acetylcholine (ACh, 10 μmol/l). ACh-induced relaxation of the precontracted rings was more than 80% in all cases indicating that the endothelium was functionally intact. After several washouts, the mesenteric arteries were again contracted with phenylephrine (0.1–3 μmol/l) and cumulative concentration-response curves to the endothelium-dependent relaxant agonist, ACh (0.1 nmol/l-10 μmol/l) and the endothelium-independent relaxant sodium nitroprusside (SNP, 0.01 nmol/l-10 μmol/l) were examined. Furthermore, after 20 minutes, responses to ACh and SNP were investigated with different combinations of N-nitro-L-arginine (L-NNA, 100 μmol/l), an inhibitor of nitric oxide synthase (NOS), 1H-[1,2,4]oxadiazolo[4,3-a]quinoxalin-1-one (ODQ, 10 μmol/l), an inhibitor of soluble guanylate cyclase (sGC), apamin (1 μmol/l), a selective blocker of the small conductance calcium-activated K^+^ channel (SK_Ca_), 1-[(2-chlorophenyl) (diphenyl)methyl]-1H-pyrazole (TRAM-34, 1 μmol/l), a blocker of the intermediate conductance calcium-activated K^+^ channel (IK_Ca_), and iberiotoxin (Ibtx, 100 nmol/l), a blocker of the large conductance calcium-activated K^+^ channel (maxi K_Ca_ or BK_Ca_).

### Estimation of basal release of NO

In order to investigate the effect of diabetes on the basal level of NO release, endothelium- intact blood vessels were exposed to L-NNA (100 μmol/l) after precontraction with phenylephrine (10–100 nmol/l) to ~ 20% KPSS. A contractile response to L-NNA was considered to indicate inhibition of basal release of NO.

### Western blots

Western blots were performed by collecting the mesenteric arteries from normal and diabetic rats. From the same treatment group, the endothelium-intact small mesenteric arteries from 2 animals were pooled and considered as n = 1. Thus the group western blot data where n = 6 is derived from arteries from 12 animals. The same amount of protein homogenate of the treatment animal groups were exposed to SDS-PAGE and analyzed through western blot processes with mouse/rabbit primary antibodies (all 1:1000, overnight, 4°C) including endothelial NO synthase (eNOS), Nox2 (all BD Transduction Laboratories, Lexington, KY, USA). The membranes were reinvestigated with a loading control antibody (actin), in order to normalize for the amount of protein. After 1 hour incubation at room temperature with anti-mouse/rabbit secondary antibody (1:2000) (Millipore, Billerica, MA, USA), all proteins were identified by enhanced chemiluminescence reagents (Amersham, GE Healthcare, Sydney, NSW, Australia). Densitometry (Biorad Chemidoc, Sydney, NSW, Australia) was used to quantify the protein bands expressed as a ratio of the loading control. To examine tissue eNOS ratio of monomer/dimer formation, 6% SDS-PAGE at 4°C used to resolve a non-boiled sample and the membranes were examined and imaged as described above.

### Measurement of superoxide release

To measure superoxide generation in mesenteric arteries, lucigenin-enhanced chemiluminescence was used. The superior mesenteric artery was isolated, cleared of fat and connective tissue and incubated for 45 minutes at 37°C in Krebs- HEPES buffer solution containing NADPH (100 μmol/l) as a substrate for NADPH-oxidase in the presence or absence of diphenyliodonium (DPI, 5 μmol/l), a flavoprotein inhibitor that inhibits NADPH oxidase. 300 μl of Krebs-HEPES buffer, containing lucigenin (5 μM) and the different treatments were placed into a 96-well Optiplate. The Optiplate was loaded into a Polarstar Optima photon counter (BMG Labtech, Melbourne, VIC, Australia) to measure background photon emission at 37°C. After the background reading was recorded, a single ring section of mesenteric artery was placed into each well and photon emission was recounted. The mesenteric arteries were placed in Krebs-HEPES buffer that contain either lucigenin (5 μM) alone or with the presence of NADPH (100 μM) to measure the production of NADPH oxidase- driven superoxide. The signal stimulated by NADPH was measured in the presence or absence of DPI (5 μM). The background counts were subtracted from superoxide counts and normalized with dry tissue weight.

### Materials

Linagliptin was a gift from (Boehringer Ingelheim, Germany). All other chemicals were purchased from Sigma-Aldrich (St Louis, MO, USA), except for ODQ (Cayman Chemical, Ann Arbor, MI, USA), and acetylcholine perchlorate (BDH Chemicals, Poole, Dorset, UK). All drugs were dissolved in distilled water with the exception of indomethacin, which was dissolved in 0.1 M sodium carbonate, and L-NNA, which was dissolved in 0.1 M sodium bicarbonate.

### Statistical analysis

Data were expressed as mean ± SEM, *n* indicates the number of experiments. The concentration response data from rat isolated mesenteric arteries were fitted to a sigmoidal curve using nonlinear regression (Prism version 6.0, GraphPad Software, San Diego, CA, USA) to calculate the pEC_50_. The maximum relaxation (R_max_) to ACh or SNP was determined as a percentage of the phenylephrine precontraction. Differences between groups of pEC_50_ and R_max_ values were compared and analysed using two way ANOVA with *post hoc* analysis for multiple comparisons using Tukey’s test. P<0.05 was considered statistically significant.

## Results

### Body weights and blood glucose

**Fig 1:**
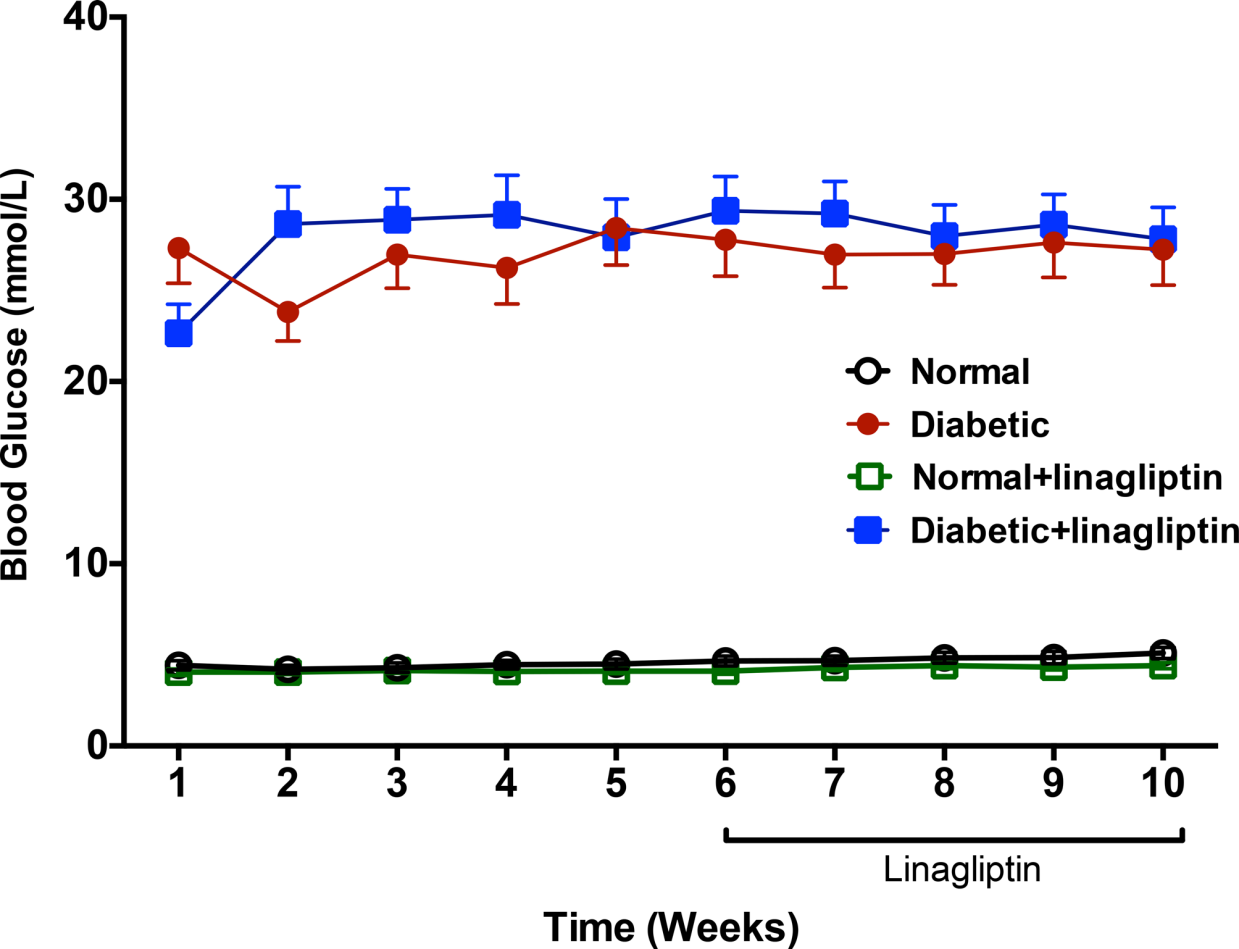
Plasma glucose concentrations measured from 1–10 weeks after administration of STZ (50 mg/kg IV). Linagliptin treatment (2 mg/kg po per day) commenced 6 weeks after STZ administration. Linagliptin did not affect plasma glucose in normal or STZ treated rats.

Blood glucose over the 10 week treatment period is shown in Fig 1 and body weight, blood glucose and HbA_1c_ levels of rats at the time of tissue collection in the 4 groups are shown in Table 1. Ten weeks after treatment with streptozotocin or vehicle, the body weight in normal rats was significantly higher than in diabetic rats (Table 1). Furthermore, in diabetic rats, the blood glucose and HbA1c levels were significantly greater than in normal rats. Four weeks treatment with linagliptin had no significant effect on body weight, HbA1c or blood glucose levels in either normal or diabetic rats.

**Table 1:** Mean body weight, blood glucose and HbA_1c_ levels at the end of the experiment of normal and diabetic rats with or without treatment with linagliptin (2 mg/kg oral gavage daily for 4 weeks) 10 weeks after vehicle or STZ treatment.

	Control	n	Control + Linagliptin	n	Diabetic	n	Diabetic + Linagliptin	n
**Body weight (g)**	498±18^b^	8	538±21^b^	9	346±11^a^ ^,^ ^c^	11	379±25^a^ ^,^ ^c^	11
**Blood glucose (mM)**	5.1±0.3^b^	8	4.5±0.2^b^	8	26.4±2^a^ ^,^ ^c^	11	27.8±1.7^a^ ^,^ ^c^	12
**HbA**_**1c**_ **(%)**	6.2±0.2^b^	7	5.8±0.2^b^	8	14.8±0.6^a^ ^,^ ^c^	10	13.9±0.4^a^ ^,^ ^c^	12

n = the number of rats.

^a^ Significantly different to normal group, p<0.05, Tukey’s test

^b^ Significantly different to diabetic group, P<0.05, Tukey’s test

^c^ Significantly different to normal+linagliptin group, P<0.05, Tukey’s test.

Results are shown as mean±SEM.

### Effect of linagliptin on vascular superoxide production

**Fig 2:**
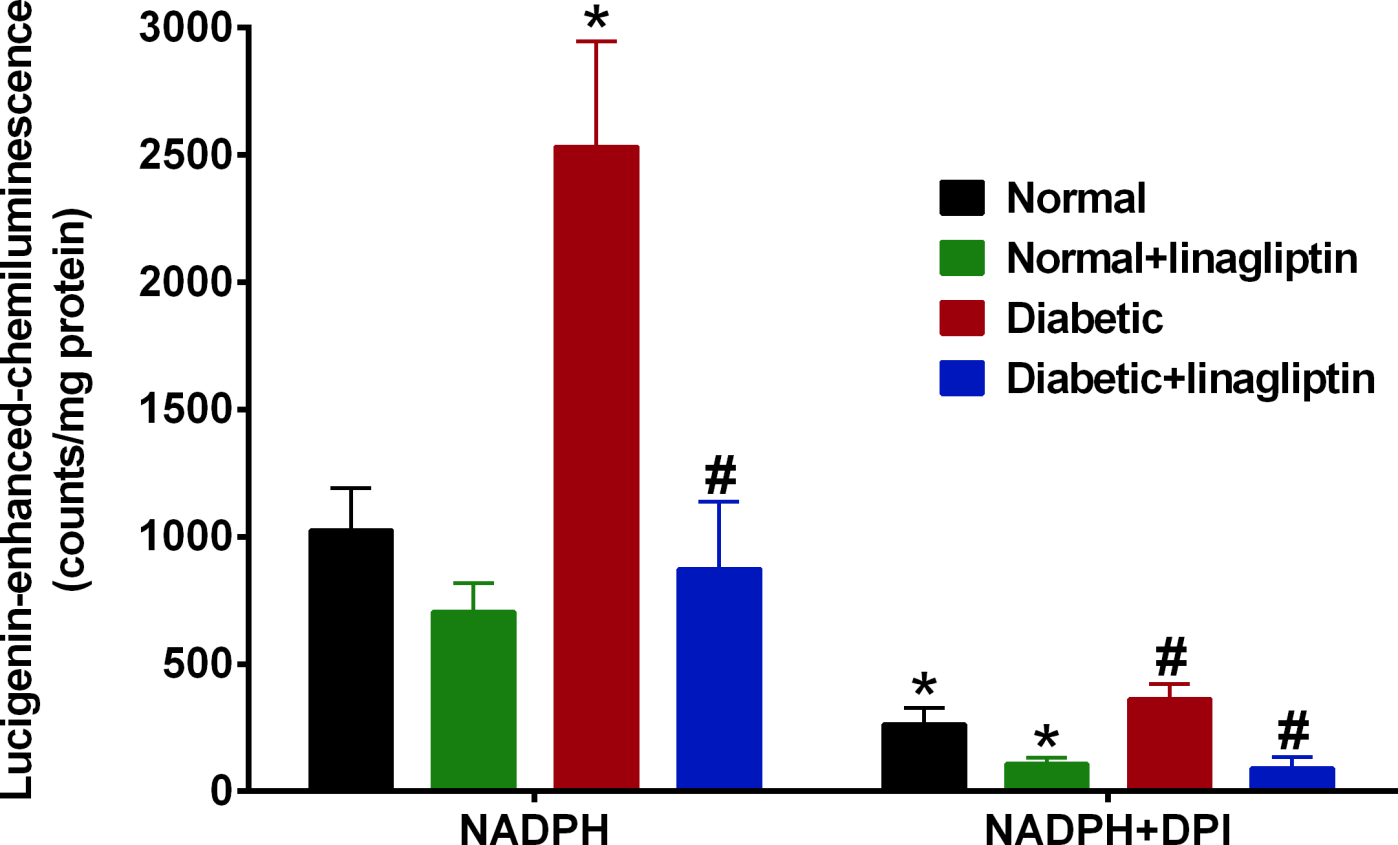
ROS measurement in intact mesenteric arteries. NADPH activity was elevated in diabetic mesenteric arteries and this was attenuated by linagliptin treatment or by DPI (5 μM), a flavoprotein inhibitor that inhibits NADPH oxidase. Results are shown as mean±SEM. n = 7–10 experiments. *P<0.05 vs normal, ^#^P<0.05 vs diabetic.

The superoxide release by mesenteric arteries was measured using lucigenin-enhanced chemiluminescence. In diabetic rats, the level of superoxide-induced fluorescence was significantly higher than that in normal rats (Fig 2). After 4 weeks of treatment with linagliptin, the level of NADPH oxidase-driven superoxide was significantly attenuated in diabetic rats, but there was no effect in normal rats (Fig 2). In all groups, diphenyliodonium was able to inhibit NADPH oxidase-driven superoxide production in the mesenteric arteries (Fig 2).

#### Effects of diabetes and linagliptin on vascular function

**Fig 3:**
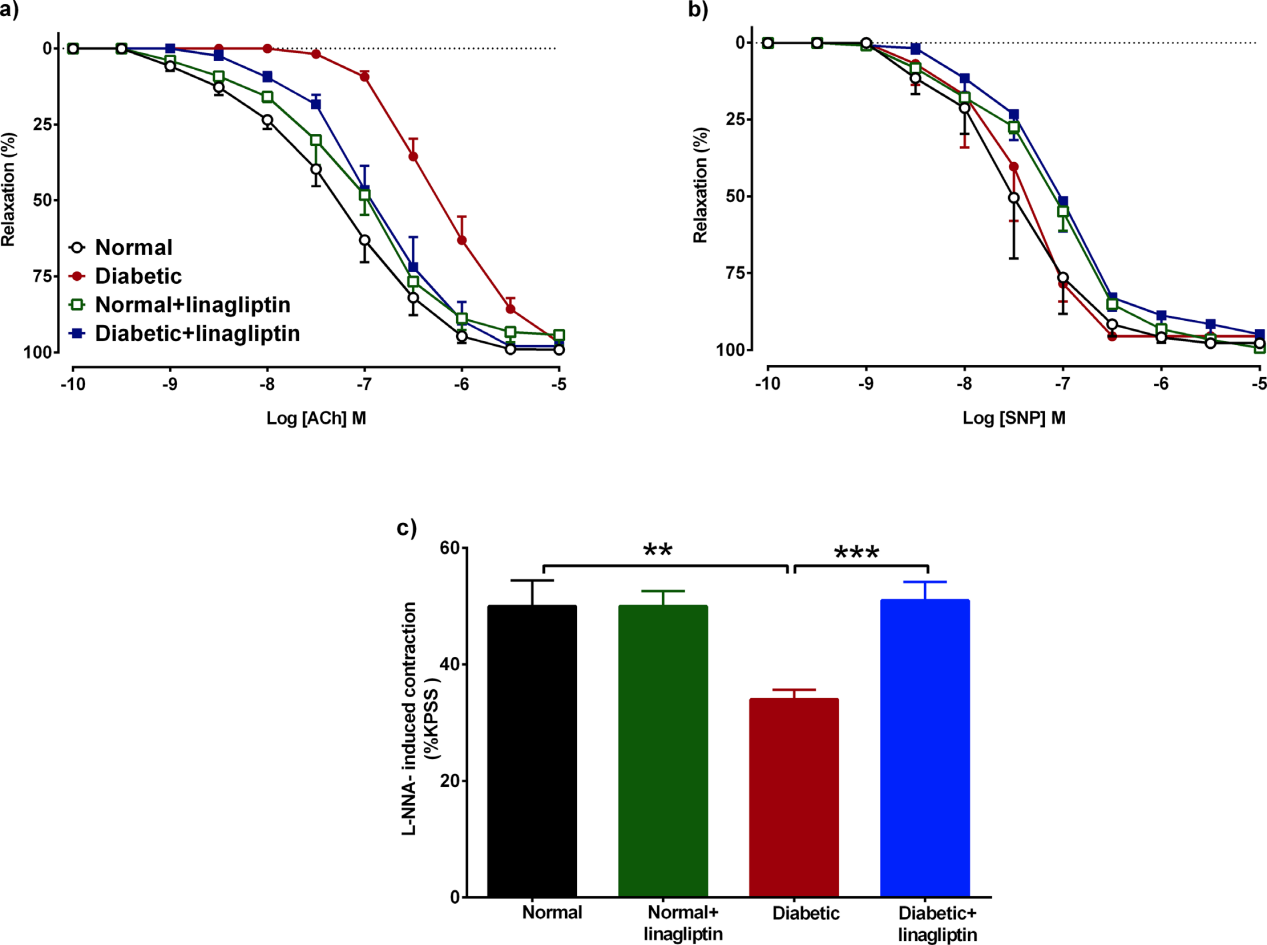
Cumulative concentration-response curves to ACh (a), SNP (b), and basal NO release (c) in endothelium-intact mesenteric arteries. In each group (a, b), mesenteric arteries were prcontracted with PE to a similar levels: (a) normal 66±2, normal+linagliptin 64±1, diabetic 65±1, diabetic+linagliptin 66±1, (b) normal 64±1, normal+linagliptin 64±1, diabetic 61±6, diabetic+linagliptin 66±1%KPSS, n = 7–10 experiments. Results are shown as mean±SEM. **P<0.01, ***P<0.001 See Table 2 or results section for pEC_50_ and R_max_ values derived from this data.

Neither diabetes nor linagliptin treatment had any effect on the level of contraction to the high K^+^ physiological saline solution (KPSS, KCl 123 mmol/l, data not shown). The sensitivity, but not the maximum relaxation, to ACh was significantly reduced in mesenteric arteries from diabetic rats (Fig 3A, Table 2), whereas the sensitivity and the maximum relaxation to sodium nitroprusside (SNP) were not affected (Fig 3B). Four weeks of linagliptin treatment (2 mg/kg per day, oral gavage) did not affect the response to ACh in mesenteric arteries from normal rats, but in diabetic rats treated with linagliptin, the sensitivity to ACh was significantly increased in comparison to the sensitivity and response to ACh in mesenteric arteries from untreated diabetic rats (Fig 3A, Table 2). The treatment with linagliptin had no effect on the response to SNP in normal or diabetic rats (Fig 3B).

**Table 2:** Effect of L-NNA, ODQ and potassium channel blockers on ACh-induced relaxation of mesenteric arteries from normal and diabetic rats with or without linagliptin (2 mg/kg oral gavage daily for 4 weeks) treatment in the presence of indomethacin.

	Normal			Normal+linagliptin			Diabetic			Diabetic+linagliptin		
ACh	pEC_50_ (M)	R_max_ (%)	n	pEC_50_ (M)	R_max_ (%)	n	pEC_50_ (M)	R_max_ (%)	n	pEC_50_ (M)	R_max_ (%)	n
**Control**	7.26±0.13	99±0	8	7.02±0.12	94±3	9	6.14±0.11^b^ ^,c^	96±1	10	6.84±0.13^d^	97±0	10
**TRAM34+apamin**	6.52±0.06^a^	91±2	7	6.29±0.10^a^	95±1	7	6.08±0.14	35±9^a^ ^,^ ^b^ ^,^ ^c^	9	6.59±0.18	96±2^d^	9
**L-NNA+ODQ**	6.31±0.09^a^	95±0	7	6.45±0.09	94±0	7	5.59±0.05^b^ ^,^ ^c^	81±7	8	6.38±0.10^d^	95±0	8
**LNNA+ODQ+ TRAM-34+apamin**	5.62±0.29^a^	27±8^a^	7	5.35±0.21^a^	45±7^a^	4	5.45±0.06^a^	4±^a^ ^,^ ^b^ ^,^ ^c^	4	5.35±0.11^a^	2±1^a^ ^,^ ^b^ ^,^ ^c^	7
**LNNA+ODQ+ TRAM-34+apa+Ibtx**	5.27±0.17^a^	14±2^a^	4	5.31±0.07^a^	25±8^a^	4	ND			ND		

A comparison of the sensitivity (pEC_50_) and maximum relaxation (R_max_) to ACh in the absence (control), or the presence of TRAM-34 (1 μM)

+apamin (1 μM), L-NNA (100 μM)+ODQ (10 μM), L-NNA (100 μM)+ODQ (10 μM)+ TRAM-34 (1 μM) +apamin (1 μM) or L-NNA (100 μM)

+ODQ (10 μM)+TRAM-34 (1 μM) +apamin (1 μM)+Ibtx (100 nM) in endothelium intact mesenteric arteries. All experiments were performed in the presence of indomethacin (10 μM). n = the number of experiments.

^a^ Significantly different to control within each group, P<0.05, Tukey’s test

^b^ Significantly different to normal within inhibitor group, P<0.05, Tukey’s test

^c^ Significantly different to normal+linagliptin within inhibitor group, P<0.05, Tukey’s test

^d^ Significantly different to diabetic within inhibitor group, P<0.05, Tukey’s test. Results are shown as mean±SEM

ND = not determined.

The basal level of NO release was investigated by assessing the level of contraction induced by L-NNA in mesenteric arteries pre-contracted with PE to 20% of the KPSS response. In normal mesenteric arteries the L-NNA- induced contraction was significantly greater than that in diabetic mesenteric arteries (Fig 3C) showing that diabetes reduced the basal release of NO. Treatment with linagliptin significantly increased the L-NNA-induced contraction in diabetic mesenteric arteries compared to untreated diabetic arteries but had no effect on L-NNA-induced contraction in normal mesenteric arteries (Fig 3C).

### Determination of the contribution of NO and EDH to endothelium-dependent relaxation

**Fig 4:**
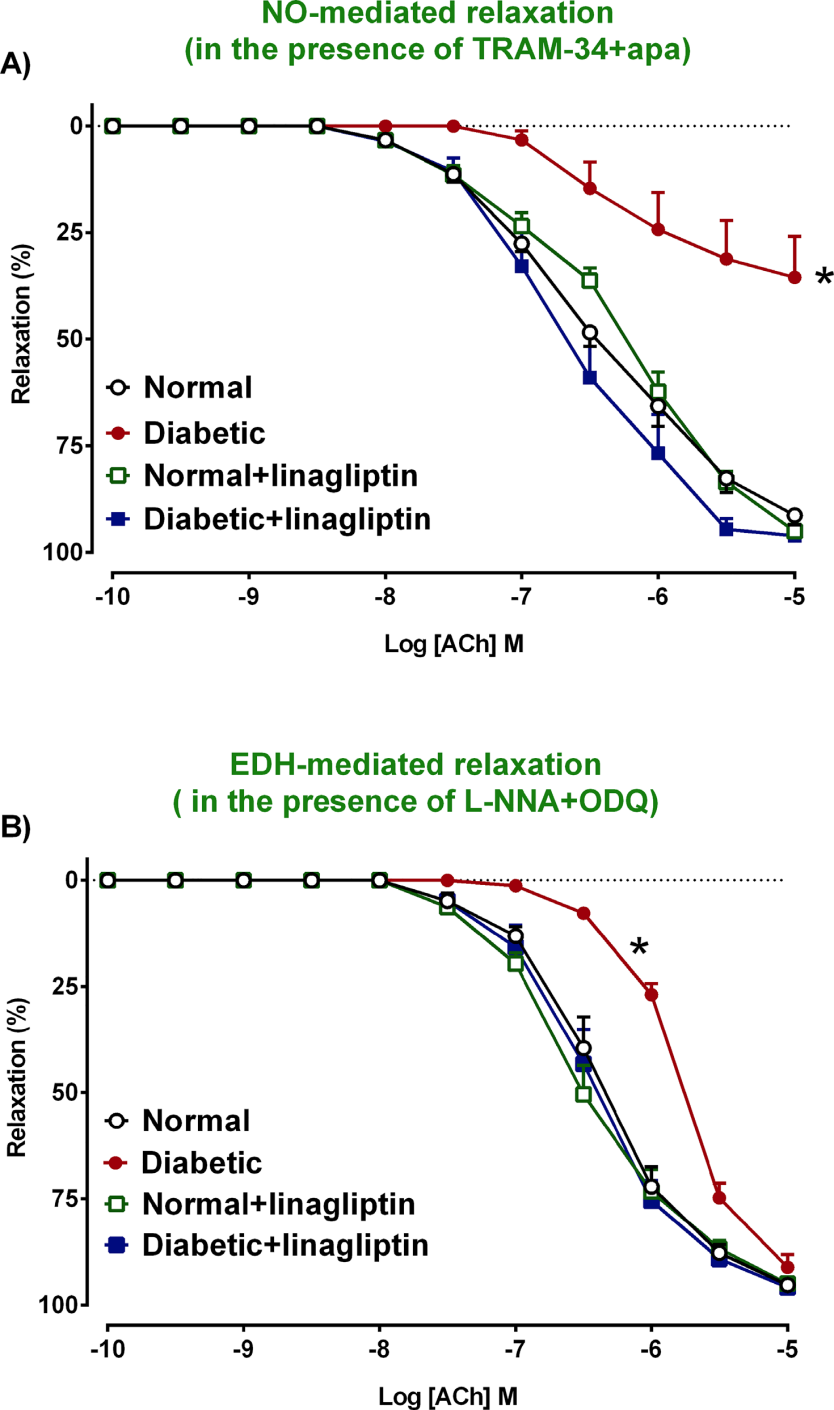
Relative contribution of NO and EDH to endothelium-dependent relaxation. NO and EDH-mediated relaxation in isolated mesenteric arteries from normal (a), diabetic (b), normal+linagliptin (c), diabetic+linagliptin (d) rats. In each group of experiments, arteries were precontracted with PE to similar levels: 63±2 (a), 65±0.6 (b), 65±1 (c), 63±1 (d) %KPSS, n = 8–9 experiments. Results are shown as mean±SEM. See Table 2 for pEC_50_ and R_max_ values derived from this data. *P<0.05 vs normal.

In mesenteric arteries from normal rats, the ACh-induced relaxation was partially reduced by either the combination of N-nitro-L-arginine (L-NNA), a NO synthase inhibitor and 1H-[1,2,4]oxadiazolo[4,3-a]quinoxalin-1-one (ODQ), a soluble guanylate cyclase inhibitor or inhibitors of intermediate-conductance calcium-activated K^+^ channel (IK_Ca_), small-conductance calcium-activated K^+^ channel (SK_Ca_) with 1-[(2 chlorophenyl)(diphenyl)methyl]1H pyrazole (TRAM-34), apamin and iberiotoxin (Ibtx) respectively, confirming that both NO and EDH were involved in endothelium-dependent relaxation (Fig 4A and 4B, Table 2). Further, the response to ACh-induced relaxation in mesenteric arteries from diabetic rats was significantly further reduced in the presence of either NO inhibitors or EDH inhibitors in comparison to normal rats (Table 2), indicating that the contribution of both NO and EDH to endothelium-dependent relaxation were impaired by diabetes.

### Effect of linagliptin on NO- and EDH-mediated relaxation in the mesenteric arteries

The response to ACh was assessed in the presence of TRAM-34+apamin in order to investigate the contribution of NO to relaxation (Fig 4A). Treatment with linagliptin for 4 weeks (2 mg/kg, daily oral gavage), significantly increased the maximum relaxation to ACh in mesenteric arteries from diabetic rats but had no effect on responses in normal rat arteries (Table 2) indicating that linagliptin could restore the contribution of NO to endothelium-dependent relaxation in diabetes.

To investigate the contribution of EDH-mediated relaxation, the actions of NO were inhibited by the addition of L-NNA and ODQ (Fig 4B). Treatment of normal and diabetic rats for 4 weeks with linagliptin (2 mg/kg, daily oral gavage) significantly increased on ACh-induced EDH mediated relaxation diabetic arteries but not in arteries from normal rats (Table 2).

### Effect of linagliptin on Nox2, total-eNOS and eNOS monomer/dimer

**Fig 5:**
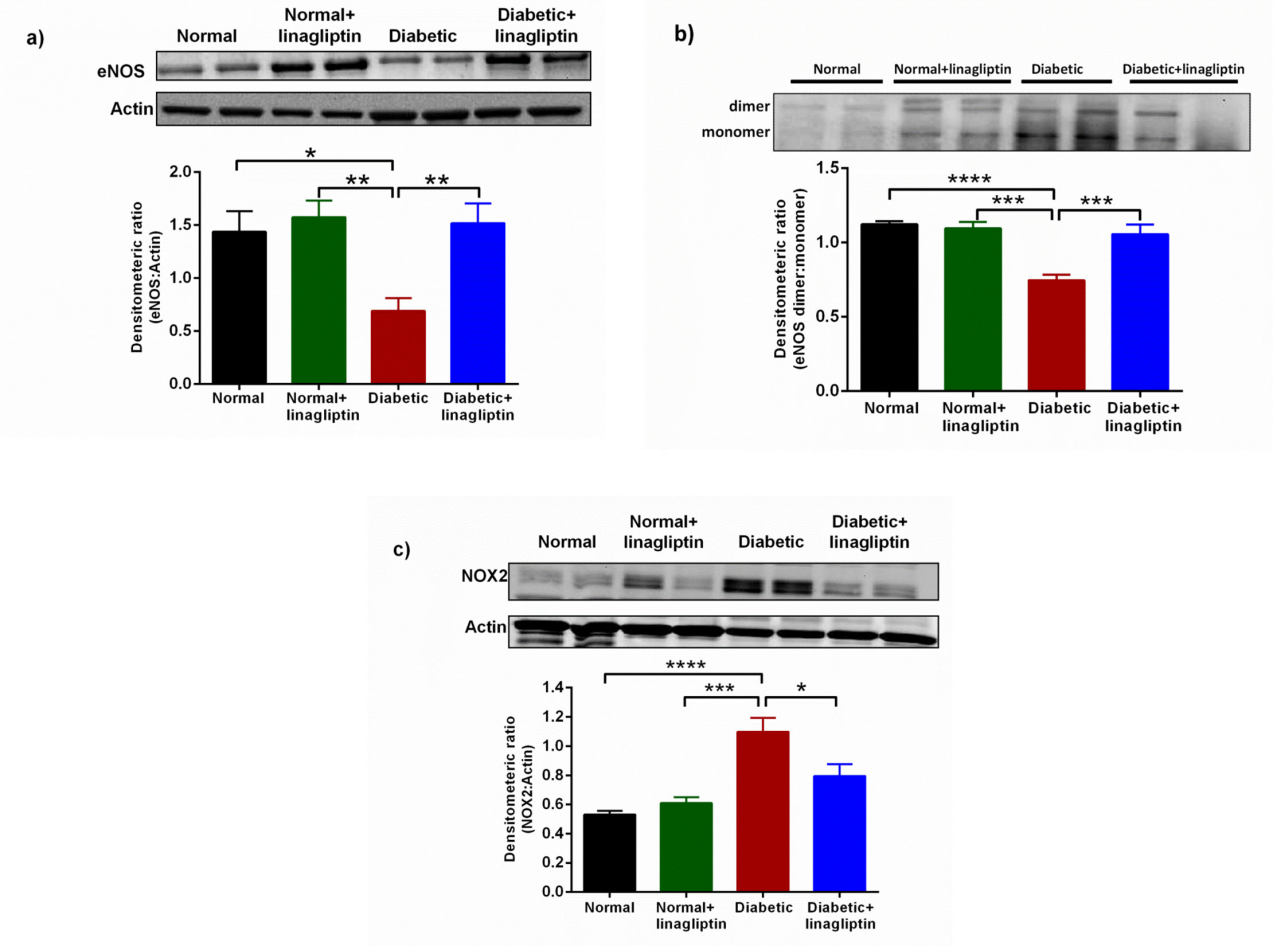
Western blot analysis of protein expression of eNOS (a, 130 kDa), eNOS dimers and monomers (b, 260 kDa) and Nox2 Nox2 (c, 58 kDa) in the normal and diabetic mesenteric arteries with or without linagliptin treatment. In diabetic mesenteric arteries, the expression of eNOS significantly reduced and the proportion of eNOS expressed as the dimer reduced, and the expression of Nox2 increased. Treatment with linagliptin increased the expression of eNOS significantly and reduced Nox2 expression and increased the proportion of eNOS expressed as the dimer. Representative blots are shown on each of the corresponding graphs. n = 6 experiments. Results are shown as mean±s.e.m. *P<0.05, **P<0.01, ***P<0.001, ****P<0.0001.

In diabetic rats, the expression of total eNOS in the mesenteric arteries was significantly reduced as was the proportion of eNOS expressed as the dimer and the expression of Nox2 was significantly increased (Fig 5A, 5B and 5C). Treatment with linagliptin did not affect the expression or dimerization of eNOS nor the expression of Nox2 in normal rats. By contrast, in diabetic mesenteric arteries, after linagliptin treatment there was a significant increase in eNOS, both in total and as a dimer, and a decrease in Nox2 expression (Fig 5A, 5B and 5C).

## Discussion

In the present study, treatment of STZ-induced type 1 diabetic rats with the DPP-4 inhibitor linagliptin (2 mg/kg, daily oral gavage) for 4 weeks decreased the levels of vascular oxidative stress and improved endothelium-dependent relaxation in mesenteric arteries. Importantly in this type 1 diabetes model the beneficial vascular effects of linagliptin were achieved without affecting plasma concentrations of glucose or HbA_1c_ demonstrating that linagliptin has actions in addition to the capacity to lower plasma glucose. A similar dose of linagliptin (1 mg/kg po) to that used in this study (2 mg/kg po) has been demonstrated to effectively inhibit DPP-4 activity and to increase GLP-1 in Zucker diabetic fatty rats [24] but in this type 1 model of diabetes, where the beta pancreatic cells are destroyed by necrosis, it is not possible to elevate insulin secretion to decrease glucose. The dysfunction in the mesenteric arteries of diabetic rats involved impairment of the contribution of both NO and EDH-mediated relaxation to endothelium-dependent relaxation. Treatment of diabetic rats with linagliptin improved the contribution of both NO and EDH to endothelium-dependent relaxation, effects which were associated with an increased expression of total eNOS, improved eNOS dimerization and reduced vascular Nox2 expression.

In the present study, STZ-induced type 1 diabetes increased the level of vascular ROS generation concomitant with a selective impairment of endothelium-dependent relaxation and an increase in the expression of Nox2 in the mesenteric arteries consistent with previous reports [7]. In diabetic rats, the *in vivo* treatment with the DPP-4 inhibitor linagliptin for 4 weeks decreased the generation of superoxide associated with decreased expression of Nox2 and improved coupling of eNOS, demonstrated by an increased ratio of eNOS dimer/monomer, in the mesenteric arteries of diabetic rats. Our observations are similar to observations that treatment with the linagliptin protects against cardiovascular injury induced by salt-sensitive hypertension via a reduction of oxidative stress [25] or improves endothelial function and reduces vascular stress in experimental sepsis in large conduit aortic artery [20]. We [8] and others [20] have previously demonstrated that linagliptin is able to exert antioxidant activity independently of any reduction in glucose, an action that was not shared by 2 other DPP-4 inhibitors sitagliptin and vildagliptin [8]. Our study [8] demonstrated that linagliptin could reduce elevated superoxide levels caused by acute exposure to the auto-oxidant pyrogallol and high glucose suggesting a radical scavenging action. It is also known that linagliptin can inhibit xanthine oxidase [26] a well known generator of superoxide. It has been reported that oxidative stress increased NO inactivation in vascular endothelium via enhanced formation of superoxide impaired eNOS activity by relative deficiency of tetrahydrobiopterin (BH_4_) and increasing production of NADPH-oxidase which may react with NO, thereby enhancing the production of the NO/superoxide end product peroxynitrate, which in turn has been shown to cause eNOS uncoupling [27]. Taken together, the ROS-induced tetrahydrobiopterin depletion and increased production of peroxynitrate may contribute to endothelial dysfunction noted in the present study and linagliptin by reducing oxidative stress may improve the coupling of eNOS that we observed. Further the inhibition of DPP-4, and consequent increase in GLP-1 and GIP, may have other actions on endothelial function that remain to be identified.

The findings in the present study indicate that there was an impairment in endothelium-dependent relaxation in small resistance mesenteric arteries in diabetic rats and this is in agreement with previous studies [7, 28]. Treatment of diabetic rats in this study with linagliptin improved responses to ACh-induced relaxation in mesenteric arteries, this may be in part by preventing the generation of superoxide anions via the antioxidant effect of linagliptin [8] and reducing eNOS uncoupling and improving the bioavailability of NO in vascular endothelial cells as we found in this study. Additionally, SNP was used to investigate the effect of diabetes on vascular smooth muscle relaxation. The response to SNP in this study was similar in normal and diabetic rats, indicating that the response to exogenous NO-induced relaxation in smooth muscle cell was not impaired by hyperglycaemia. It has previously been reported that STZ-induced diabetes causes a progressive impairment of endothelial function [29] and we have reported disruption of the mechanisms of endothelium dependent relaxation 6 weeks after STZ treatment [30]. Kitayama et al. (2006) reported that, in cerebral arterioles, responses to the endothelium-dependent agonist acetylcholine were normal 3 weeks after STZ but impaired at 5–6 weeks [31]. In this study as the rats were diabetic for 6 weeks before linagliptin treatment was instigated it seems likely that there was some dysfunction before the treatment commenced but equally dysfunction was likely to progress over the next 4 weeks. Whilst it seems likely that linagliptin was able to reverse as well as to prevent progression of endothelial dysfunction further experiments are required to confirm that possibility.

We and others have previously reported that both NO and EDH are important mediators of endothelium-dependent relaxation of rat mesenteric arteries [8] and that both mechanisms of relaxation are impaired by diabetes [7]. We therefore investigated whether linagliptin affected the contribution of NO, EDH or both to endothelium-dependent relaxation. In order to investigate NO-mediated relaxation, the EDH-mediated relaxation was blocked by endothelial K_Ca_ channel blockers. Where NO mediated relaxation was blocked, the maximum relaxation to ACh was significantly reduced in the mesenteric arteries from diabetic rats in comparison to normal rats verifying that diabetes impaired the contribution of NO to endothelium-dependent relaxation. Chronic treatment with linagliptin *in vivo* for 4 weeks significantly increased the NO mediated maximum relaxation to ACh in diabetic mesenteric arteries in comparison to arteries from untreated diabetic rats. Furthermore, in addition to stimulated NO release, basal NO release was also reduced in mesenteric arteries of diabetic rats. This was demonstrated by a reduced contractile response to the NOS inhibitor L-NNA in diabetic arteries but this was not observed in arteries from linagliptin treated diabetic rats. Thus, treatment with linagliptin preserved the contribution of NO to endothelium-dependent relaxation in diabetic mesenteric arteries. There are several mechanisms that may have been affected by linagliptin to improve the level of activity of NO in diabetes. Firstly there was an increase in expression and dimerization of eNOS when diabetic rats were treated with linagliptin which would result in an increase synthesis of NO. Further the bioactivity of NO may be increased secondary to the decrease of vascular superoxide generation in diabetic mesenteric arteries, reducing the formation of peroxynitrite (ONOO^-^) via the degradation of NO by superoxide anions. The reduction of ROS activity in the diabetic vasculature could be due to a free radical scavenging effect of linagliptin such as we previously observed in vitro [8]. As we also observed a reduction in the expression of Nox2 there may also have been a reduced level of vascular NADPH oxidase activity contributing to the reduction in oxidative stress. linagliptin, through the inhibition of xanthine oxidase [26] Taken together, chronic treatment with linagliptin preserved the beneficial activity of NO which was associated with improving eNOS expression, as well as re-coupling of eNOS and reduced expression of Nox2, which mediate superoxide generation in the mesenteric arteries of diabetic rats.

The endothelium-dependent relaxation in rat mesenteric arteries is mediated by classical EDH and non-classical EDH pathways [32] in addition to NO. In order to evaluate the contribution of EDH to endothelium-dependent relaxation in diabetes, we investigated the endothelium-dependent relaxation in the presence of L-NNA and ODQ to inhibit NO formation and sGC activity respectively. The guanylate cyclase inhibitor was also used to confirm the inhibition of the action of NO derived from nitrosothiols, a non-NOS source of NO, which we have previously reported to act as a source of NO in diabetic conditions [33]. The sensitivity to ACh in diabetic mesenteric arteries was decreased significantly in comparison to normal mesenteric arteries in the presence of L-NNA+ODQ, indicating that diabetes impaired the contribution of EDH to endothelium-dependent relaxation, which is consistent with other reports [7, 34–36]. Chronic *in vivo* treatment with linagliptin improved EDH-mediated relaxation in mesenteric arteries of diabetic rats, indicating a preserved contribution of EDH to endothelium-dependent relaxation. Although the main cause of the impairment of EDH in diabetic conditions remains uncertain [7, 35, 37–40], it is associated with oxidative stress. It is reported that auto-oxidation of pyrogallol causes an increase in superoxide anions which could impair EDH-mediated relaxation in rat mesenteric arteries [41] and our results demonstrated that the impairment of EDH-mediated relaxation in diabetes may be in part due to the overproduction of superoxide and treatment of diabetic rats with lingaliptin improved the contribution of EDH to endothelium-dependent relaxation in mesenteric arteries.

In conclusion, in this study we have demonstrated that the DPP-4 inhibitor linagliptin improves endothelial function in mesenteric arteries in diabetic rats by preserving both NO and EDH-mediated relaxation. The DPP-4 inhibitor linagliptin was able to reduce the generation of superoxide which may be due to direct radical scavenging action and/or attenuating the enzymatic source for superoxide generation where the chronic treatment of diabetic rats with linagliptin *in vivo* for 4 weeks prevents the uncoupling of eNOS, increases the expression of total eNOS, and decreases Nox2. Thus, the ability of linagliptin to exert an antioxidant effect as we have previously reported [8] can be expressed when the drug is administered orally over a 4 week period to rats with established type 1 diabetes. It is also possible that inhibition of DPP-4 and elevation of incretins may have unreported beneficial actions on endothelial function that need to be investigated. Interestingly, the beneficial effects of linagliptin to preserve NO activity and improve EDH role in relaxation demonstrated in this study displays that linagliptin has the ability to act as a therapeutic compound independent of it is glucose lowering action for use in the prevention of the microvasculature complications of diabetes. Such activity may extend the use of linagliptin beyond the treatment of type 2 diabetes to include other vascular pathologies involving oxidative stress such as occurs in type 1 diabetes and atherosclerosis.

## Abbreviations

DPP-4: dipeptidyl peptidase-4
GIP: glucose-dependent insulinotropic polypeptide
GLP-1R: glucagon-like peptide-1 receptor
DPI: diphenyliodonium
eNOS: endothelial nitric oxide synthase
EDH: endothelium-dependent hyperpolarization
SK_Ca_: small-conductance calcium-activated K^+^ channel
IK_Ca_: intermediate-conductance calcium-activated K^+^ channel
L-NNA: N-nitro-L-arginine
BK_Ca_: large-conductance calcium-activated K^+^ channel
NO: nitric oxide
ODQ: 1H-[1,2,4]-oxadiazolo[4,2-a]quinoxalin-1-one
pEC_50_: negative logarithm of the half maximal effective concentration
R_max_: maximum relaxation
ROS: reactive oxygen species
sGC: soluble guanylate cyclase
SNP: sodium nitroprusside
TRAM-34: 1-[(2-chlorophenyl)(diphenyl)methyl]-1H-pyrazole
Ibtx: iberiotoxin
